# Expression of tumor-specific antigen MAGE, GAGE and BAGE in ovarian cancer tissues and cell lines

**DOI:** 10.1186/1471-2407-10-163

**Published:** 2010-04-27

**Authors:** Shiqian Zhang, Xiaoliang Zhou, Hao Yu, Yunhai Yu

**Affiliations:** 1 The State-Key Discipline of Obstetrics and Gynecology, Department of Obstetrics and Gynecology, Qilu Hospital of Shandong University, Ji'nan 250012, China; 2 Department of Gynecology, The Affiliated Hospital of Henan University of Science and Technology, Luoyang 471003, China; 3 Department of Women tumor, Cancer Research Institute of Shandong Province, Ji'nan 250117, China; 4 Department of Obstetrics and Gynecology, the Second Hospital of Shandong University, Ji'nan 250033, China

## Abstract

**Background:**

To observe mRNA expression of tumor-specific antigen MAGE, BAGE and GAGE in epithelial ovarian cancer tissues and cell lines, to explore the relationship between gene expression and diagnosis, treatment and prognosis of ovarian cancer, and to evaluate the feasibility of their gene products as markers, and an immunotherapy target for ovarian cancer.

**Methods:**

mRNA expression of MAGE-1, MAGE-3, GAGE-1/2 and BAGE were determined by reverse transcription polymerase chain reaction (RT-PCR) in 14 cases of normal ovarian tissue, 20 cases of ovarian benign tumor specimens, 41 cases of ovarian cancer specimens, and ovarian cancer cell lines SKOV3, A2780, and COC1.

**Results:**

MAGE, GAGE and BAGE genes were not expressed in normal ovarian tissue. In benign tumors, only the MAGE gene was expressed; the expression rate of this gene in benign tumors was 15% (3/20). In ovarian cancer tissues, MAGE-1 and MAGE-3 was highly expressed, with expression rates of 53.7% (22/41) and 36.6% (15/41), while GAGE-1/2 and BAGE had relatively low expression, with rates of 26.8% (11/41) and 14.6% (6/41). In metastatic lesions of ovarian cancer, only MAGE-1 and BAGE were expressed, with expression rates of 28.6% (2/7) and 14.3% (1/7). The positive expression rates of MAGE-1 and MAGE-3 in serous cystadenocarcinoma were significantly higher than that in other types of ovarian cancer (*P *< 0.05). Gene expression rate was not correlated with menopause or lymph node metastasis. Positive expression of MAGE-1 and MAGE-3 was positively correlated with tumor differentiation and the clinical stage of the ovarian cancer. In addition, the positive expression rate of BAGE was significantly higher in ovarian cancer patients with ascites (*P *< 0.05). The mRNA expression profiles of MAGE, GAGE and BAGE in ovarian carcinoma cell lines SKOV3, A2780 and COC1 varied, but there was at least one gene expressed in each cell line.

**Conclusion:**

Tumor-specific antigen MAGE, BAGE and GAGE may play a role in the occurrence and development of ovarian cancer. These genes can be used as one of the important indicators for early diagnosis, efficacy evaluation and prognostic determination of ovarian cancer.

## Background

Cancer-testis antigen (CTA), a type of protein restrictively expressed in the testes and malignant tumors, is considered to be associated with the cell carcinogenesis. Hence, CTA is thought to be an ideal target for cancer immunotherapy and has gained extensive attention in these years [1]. MAGE, GAGE and BAGE family genes, all of which are members of CTA, are expressed not only in melanoma cells, but also in many tumor tissues. Moreover, their expression is closely related to the occurrence, development and prognosis of cancer. The antigens encoded by these genes can be recognized by the body's immune cells and can then induce the body to produce specific humoral and cellular immunity. It has been reported that MAGE, GAGE, and BAGE genes, as well as their products, could be used for molecular diagnosis and immunotherapy of tumors [2]. In recent years, the diagnosis and treatment of ovarian cancers have improved, but the long-term survival rate, especially the survival rate for advanced cases, still has not been markedly increased. Therefore, it is very important to search for tumor-specific antigen (TSA) and tumor-associated antigen (TAA) to ensure the early detection, early diagnosis and early treatment of ovarian cancer. Domestic and international scholars have conducted a great deal of research on the gene expression profiles, functions and mechanisms of MAGE, BAGE and GAGE. However, research on the role of these genes in ovarian cancer has been sparse. In the present work, the mRNA expression of MAGE-1, MAGE-3, GAGE-1/2 and BAGE was analyzed by reverse transcription polymerase chain reaction (RT-PCR), and the feasibility of their gene products as ovarian cancer markers and immunotherapy targets was also evaluated.

## Methods

### 1. Cell culture

Ovarian cancer cell lines SKOV3, A2780 and COC1 were established in China. SKOV3, COC1 and normal ovarian epithelial cells (NOEC) were provided by the Bioengineering Center of Qilu Hospital, Shandong University. Melanoma cell lines MEL526, a positive control, were kindly given by Dr. Takesako. Cells were cultured in RPMI 1640 culture medium (containing 10% fetal calf serum, 100 u/ml penicillin and 100 u/ml streptomycin) at 37°C with 5% CO_2_. The cells were collected by conventional digestion and stored at -80°C when they reached about 80% confluence.

### 2. Clinical data and tissue samples

Patients who visited the Gynecology service of Qilu Hospital, Shandong University between January 2005 and December 2008 were selected. The tissue samples were obtained at the time of surgery in the Department of Gynecology, Qilu Hospital, Shandong University. The present study was approved by the ethics committee of the Shandong University. All samples were obtained with medical-ethics approval and all patients gave informed consent. There were 14 cases of normal ovarian tissues, 20 cases of ovarian benign tumor samples, 41 cases of ovarian cancer samples and 7 cases of metastatic lesions of ovarian cancer samples. The obtained tissues were confirmed by histopathological examination and stored in liquid nitrogen at 10 min after surgery. The patients' clinical data were recorded in detail. Tumor histological grades and clinical stages were evaluated according to the pathological results after surgery. The clinical stages of ovarian cancer were based on FIGO (presented in 2000) staging criteria. Of the 41 cases of ovarian cancer, there were 18 cases with serous cystadenocarcinoma, 13 cases with mucinous cystadenocarcinoma, 6 cases with endometrial carcinoma, and 4 cases with clear cell tumors. There were 5 cases in Stage I, 9 cases in Stage II, 23 cases in Stages III and 4 cases in Stage IV. With regards to histological grading, 6 cases were in G1, 18 cases were in G2 and 17 cases were in G3. There were 15 patients with ascites and 26 patients without ascites. Lymph node metastasis occurred in 15 cases. The patients aged between 23 and 65 years, with an average age of 45.2 years.

### 3. Determination of mRNA expression of MAGE-1, MAGE-3, GAGE-1/2 and BAGE gene

Total RNA was extracted with Trizol (Shanghai Bio-Engineering Company) from various cell lines and tissue samples. A260 and A280 of total RNA were measured by UV-spectrophotometer. To synthesize the first strand of CDNA, 4 μg of RNA was reverse-transcribed with the reverse transcriptase M-MLV using Oligo dT (Shanghai Bio-Engineering Company) as the downstream primer. PCR primers were designed on different exons to ensure the specificity of amplification and to avoid genomic DNA contamination. The primers are listed in Table 1.

**Table 1:** Primers for MAGE, GAGE and BAGE.

Gene	Primer	Sequence
MAGE-1	P1	5'-ACT ACC TTC ACT CG-3'
	P2	5'-CTC CCA TCA TAC ACC TCC-3'
MAGE-3	P1	5'-TGG AGG ACC AGA GGC CCC C-3'
	P2	5'-GGA CGA TTA TCA GGA GGC CTG C-3'
GAGE-1/2	P1	5'-GAC CAA GAC GCT ACG TAG-3'
	P2	5'-CCA TCA GGA CCA TCT TCA-3'
BAGE	P1	5'-TGG CTC GTC TCA CTC TGG-3'
	P2	5'-CCT CCT ATT GCT CCT GTT G-3'
β-actin	P1	5'-AGC GAG CAT CCC CCA AAG TT-3'
	P2	5'-GGG CAC GAA GGC TCA TCA TT-3'

The PCR program was as follows. MAGE-1 and β-actin: 35 cycles of denaturation at 94°C for 45 sec, annealing at 55°C for 45 sec and extension at 72°C for 1 min; MAGE-3: 40 cycles of denaturation at 94°C for 1 min, annealing at 66°C for 1 min 15 sec and extension at 72°C for 1 min; GAGE-1,2: 30 cycles of denaturation at 94°C for 1 min, annealing at 56°C for 2 min and extension at 72°C for 3 min; and BAGE: 30 cycles of denaturation at 94°C for 1 min, annealing at 60°C for 2 min and extension at 72°C for 2 min. The PCR program was started with pre-denaturation at 94°C for 5 min and ended with a final 72°C extension for 5 min. The PCR products were separated by electrophoresis on a 1.5% agarose gel, stained with ethidium bromide and observed with UV lamp. Results were determined by quantification with a JD801 Image Analysis system 3.3 (JiangSu JEDA Science-Technology Development Co., Ltd, Nanjing). The melanoma cell lines MEL526 were a positive control, the sample without M-MLV reverse transcriptase was a negative control, and β-actin was an internal control for the RT-PCR. According to the sequences in GenBank, the length of PCR products for MAGE-1, MAGE-3, GAGE-1/2 and BAGE genes were 485 bp, 724 bp, 243 bp and 284 bp, respectively.

### 4. Statistical analysis

All analyses were performed with SPSS 10.0 software. The measurement data were analyzed with the *t*-test, while the comparison of menopause, pathological features (such as tumor histological type and clinical stage) and gene expression were analyzed with the χ^2 ^test. Statistical differences were defined as P < 0.05.

## Results

### 1. mRNA expression of MAGE, GAGE and BAGE in ovarian cancer cell lines

**Figure 1:**
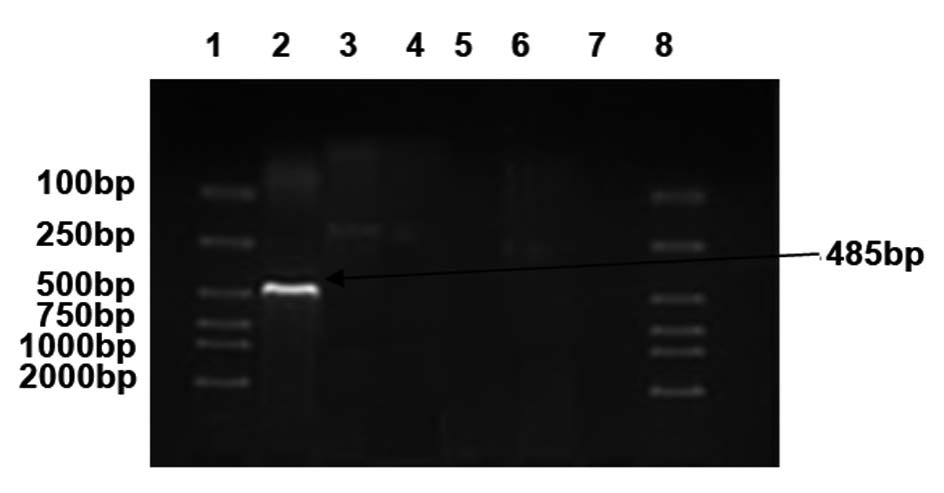
**mRNA expression profile of MAGE, BAGE and GAGE in SKOV3 cells**. (1, 8: DL 2000 marker, 2: MAGE-1, 3: MAGE-3, 4: BAGE, 5.GAGE-1/2, 6,7: negative control).

**Figure 2:**
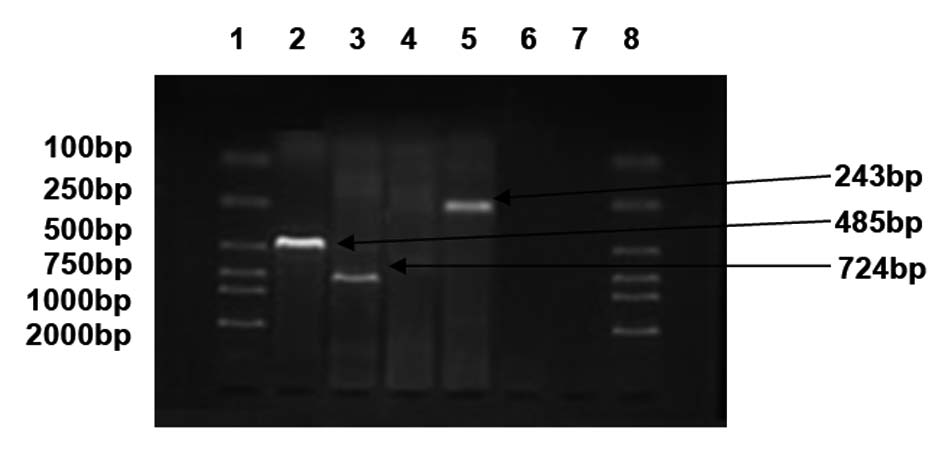
**mRNA expression profile of MAGE, BAGE and GAGE in A2780 cells**. (1, 8: DL 2000 marker, 2: MAGE-1, 3: MAGE-3, 4: BAGE, 5.GAGE-1/2, 6,7: negative control).

**Figure 3:**
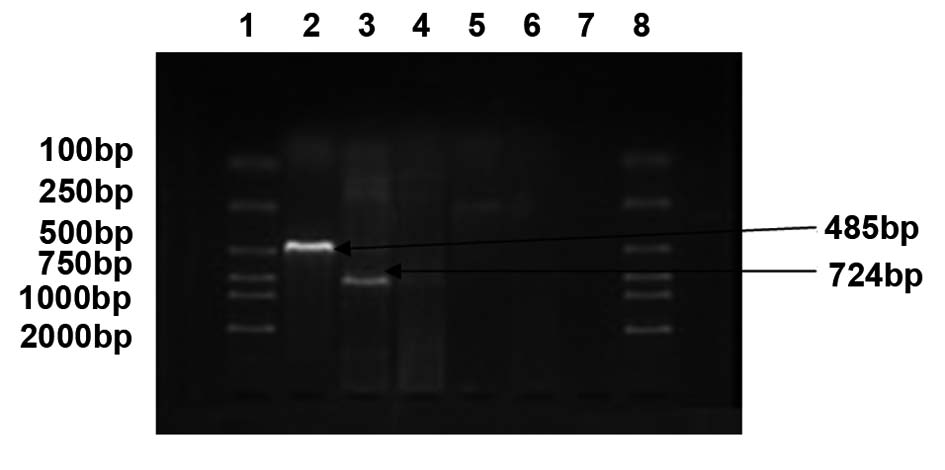
**mRNA expression profile of MAGE, BAGE and GAGE in COC1 cells**. (1, 8: DL 2000 marker, 2: MAGE-1, 3: MAGE-3, 4: BAGE, 5.GAGE-1/2, 6,7: negative control).

The mRNA expression profiles of MAGE, GAGE and BAGE in ovarian carcinoma cell lines SKOV3, A2780 and COC1 were varied, but there was at least one gene expressed in each cell line. In SKOV3 (Figure 1), MAGE-1 was expressed (line 2, a band ranging between 400-500 bp). In A2780 (Figure 2), MAGE-1 (line 2, a band ranging between 400-500 bp), MAGE-3 (line 3, a band ranging between 700-800 bp) and GAGE-1/2 (line 5, a band ranging between 200-300 bp) were expressed. In COC1 (Figure 3), MAGE (line 2, a band ranging between 400-500 bp) and MAGE-3 (line 3, a band ranging between 700-800 bp) were expressed.

### 2. mRNA expression of MAGE, GAGE and BAGE in various ovarian tissues

**Figure 4:**
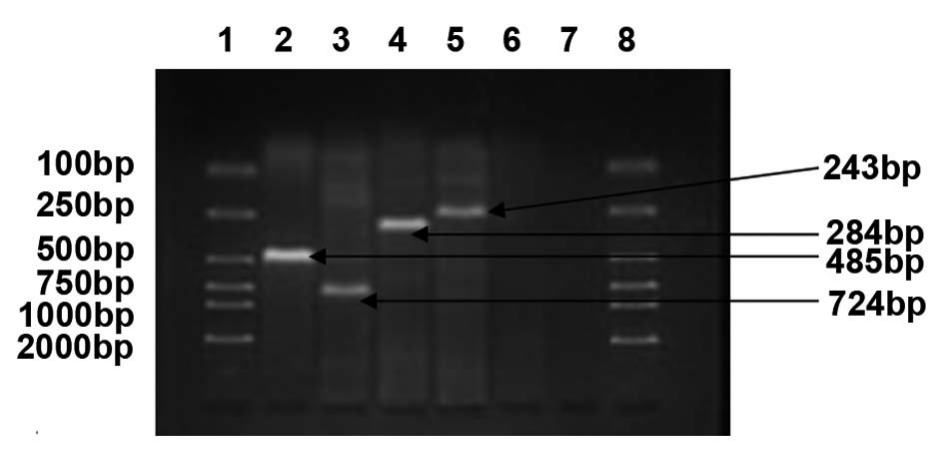
**mRNA expression profile of MAGE, BAGE and GAGE in ovarian cancer tissues**. (1, 8: DL 2000 marker, 2: MAGE-1, 3: MAGE-3, 4: BAGE, 5.GAGE-1/2, 6,7: negative control).

MAGE, GAGE and BAGE genes were not expressed in normal ovarian tissue. In benign tumors, only the MAGE gene was expressed, and the positive rate was 15% (3/20). In ovarian cancer tissues, MAGE-1 and MAGE-3 were highly expressed with expression rates of 53.7% (22/41) and 36.6% (15/41), while GAGE-1/2 and BAGE had relatively low expression rates of 26.8% (11/41) and 14.6% (6/41). In metastatic lesions of ovarian cancer, only MAGE-1 and BAGE were expressed with expression rates of 28.6% (2/7) and 14.3% (1/7) (Table 2 & Figure 4).

**Table 2:** mRNA expression of MAGE, GAGE and BAGE in various ovarian cancer tissues [Cases(%)].

Tissue	Cases (n)	MAGE-1	MAGE-3	BAGE	GAGE-1/2
Normal ovary	14	0 (0)	0 (0)	0 (0)	0 (0)
Ovarian benign tumor	20	3 (15)	0 (0)	0 (0)	0 (0)
Ovarian cancer	41	22 (53.7)	15 (36.6)	6 (14.6)	11 (26.8)
Metastatic lesions of ovarian cancer	7	2 (28.6)	0 (0)	1 (14.3)	0 (0)

### 3. The relationship between the pathological types of ovarian cancer and the positive expression rates of MAGE, GAGE and BAGE

In serous cystadenocarcinoma samples, the positive expression rates of MAGE-1 and MAGE-3 were 77.8% (14/18) and 66.7% (12/18), which were significantly higher than that in other types of ovarian cancer (*P *< 0.05). Although the expression of GAGE-1/2 and BAGE was low in ovarian cancer tissues, their positive expression rates were relatively high in serous cystadenocarcinomas (Table 3).

**Table 3:** mRNA expression of MAGE, GAGE and BAGE in different pathological types of ovarian cancer [Cases(%)].

Pathological type	Cases (n)	MAGE-1	MAGE-3	BAGE	GAGE-1/2
Serous cystadenocarcinoma	18	14 (77.8)	12 (66.7)	4 (22.2)	6 (33.3)
Mucinous cystadenocarcinoma	13	5 (38.5)	2 (15.4)	1 (7.7)	3 (23)
Endometrial carcinoma	6	2 (33.3)	1 (16.6)	0 (0)	1 (16.7)
Clear cell tumors	4	1 (25)	0 (0)	1 (25)	1 (25)

### 4. The correlation between the pathological features of ovarian cancer and the positive expression rates of MAGE, GAGE and BAGE

Clinical data of ovarian cancer patients with or without MAGE, GAGE or BAGE expression were analyzed. The expression of MAGE, GAGE and BAGE was not correlated with menopause or lymph node metastasis (*P *> 0.05). Positive expression of MAGE-1 and MAGE-3 was positively correlated with the pathological grade and clinical stage of ovarian cancer patients (*P *< 0.05), while positive expression of MAGE-1 and MAGE-3 was not related to pathological grade and clinical stage (*P *> 0.05). Additionally, ascites was correlated with the expression of BAGE, as indicated by the fact that the positive expression rate of BAGE was significantly higher in ovarian cancer patients with ascites (*P *< 0.05) (Table 4).

**Table 4:** The relationship between pathological features of ovarian cancer and the positive expression rate of MAGE, GAGE and BAGE [Cases(%)].

Pathological parameters	Cases (n)	MAGE-1	MAGE-3	BAGE	GAGE-1/2
Age					
Pre-menopausal	26	16 (61.5)	9 (34.6)	4 (15.4)	7(26.9)
Post-menopausal	15	6 (40)	6 (40)	2 (13.3)	4 (26.7)
Histological grade					
G1 ~G2	24	16 (66.7)	12(50)	3 (12.5)	6(25)
G3	17	6 (35.3)	3(17.6)	3(17.6)	5 (29.4)
Clinical stage					
I ~II	14	4(28.6)	2 (14.3)	2 (14.3)	4 (28.6)
III ~IV	27	18 (66.7)	13 (48.1)	4 (14.8)	7 (25.9)
Lymph node metastasis					
With	15	10 (66.7)	8 (53.3)	3 (20)	5 (33.3)
Without	26	12 (46.2)	7 (26.9)	3(11.5)	6 (23.1)
Ascites					
With	15	10 (66.7)	8 (53.3)	5 (33.3)	7 (46.7)
Without	26	12 (46.2)	7 (26.9)	1 (3.8)	4 (15.4)

## Discussion and Conclusions

At present, 23 members have been found in the MAGE gene family. They are located on the human X chromosome and are highly expressed in a variety of tumors. The proteins encoded by these genes contain more than 300 amino acids, and peptides processed from these proteins can bind to all types of human leukocyte antigens to form complexes, which can be recognized by autologous T-lymphocytes. The GAGE gene family contains at least eight near-source genes, which are all located on the human X chromosome. Antigen peptide YRPRPRRY encoded by the GAGE-1 gene and GAGE-2 gene could interact with HLA-Cw*0601 to form a complex, which may be recognized by activated cytotoxic T-lymphocyte [3]. In addition to the several BAGE-family members that were found previously, another five new BAGE genes have been discovered and are located adjacent to the centromere of chromosome 13 or 21. Additionally, there are nine BAGE gene segments located adjacent to the centromere of chromosomes 9, 13, 18 or 21. These genes and gene segments are highly homologous (90%-99%). Polypeptides encoded by BAGE genes contain 43 amino acids, whose AARAVFLAL fragment interacts with HLA-Cw*1601 and is then recognized by activated cytotoxic T lymphocytes.

MAGE, BAGE, and GAGE proteins are processed to peptides in cancer cells by low molecular weight polypeptide (LMP) and are then transported to the lumen of the endoplasmic reticulum by transporter associated with antigen processing (TAP). The antigen peptides interact with MHC I molecules to form complexes, which are expressed on the surface of tumor cells via the Golgi apparatus. CTL can recognize the surface complexes through T cell antigen receptor (TCR) interactions and can destroy the tumor cells through a killing mechanism. Therefore, MAGE, BAGE, and GAGE genes, as well as their products have wide application prospects in tumor-specific initiative immunotherapy [4].

Many tumor marker studies have shown that BAGE, MAGE-1/3 and GAGE-1/2 are expressed in the majority of different types of ovarian cancer, while not in the normal ovarian tissue or body fluids. Russo et al. [5] detected transcription levels of MAGE-1 and MAGE-3 in 54 patients with epithelial ovarian cancer using the RT-PCR method. Their results showed that the expression rates of MAGE-1, MAGE-3, BAGE and GAGE were 28%, 17%, 15% and 31%, respectively. Moreover, they reported that an increase in the expression rate of MAGE-1 was found with the increase in tumor clinical stage, as indicated by the fact that the expression rate of MAGE-1 in Stage III and Stage IV of ovarian cancer was 22% and 43%, respectively. Gillespie et al. [6] found that the expression rate of MAGE-1 in ovarian cancer was 56%, while the expression rates of MAGE-2, -3, -4, BAGE and GAGE were relatively low. Hofmann et al. [3] obtained ascites samples obtained by fine-needle aspiration and found that BAGE, MAGE-1, MAGE-3 and GAGE-1/2 mRNA was positively expressed in 56% (15/27), 7% (2/27), 30% (8/27) and 30% (8/27) of samples, respectively. The sensitivity was 94%; the specificity was 94%; and in one case, there was a false positive when these genes were jointly detected in ascites samples. Therefore, the detection of BAGE, GAGE, and MAGE mRNA in ovarian cancer ascites may be a potential diagnostic option.

In our study, MAGE, GAGE and BAGE genes were not expressed in normal ovarian tissue, only the MAGE-1 gene was expressed in benign tumors at a rate of 15% (3/20). In ovarian cancer tissues, MAGE-1 and MAGE-3 genes were highly expressed with a positive rate of 53.7% (22/41) and 36.6% (15/41), respectively. The expression rate of the MAGE-1 gene was slightly lower than that reported by Gillespie et al. [6]. The expression rates of the GAGE-1/2 and BAGE genes in ovarian cancer were 26.8% (11/41) and 14.6% (6/41), respectively, which were significantly higher than that reported by Gillespie et al. [6]. In cancer metastasis outside the ovary, only the MAGE-1 and BAGE gene were expressed, with positive expression rates of 28.6% (2/7) and 14.3% (1/7), respectively. The positive rates of MAGE-1 and MAGE-3 genes in serous adenocarcinoma were significantly higher than that in other types of ovarian cancer (*P *< 0.05).

Further statistical analyses of MAGE, GAGE and BAGE gene expression, as well as the clinical data from 41 cases of ovarian cancer were further conducted. Our results demonstrated that gene expression was not correlated with menopause or lymph node metastasis, and MAGE-1 and MAGE-3 gene expression was closely related to cell differentiation and cancer clinical stage (*P *< 0.05). The lower the histological grade, the later the clinical stage, and the higher the expression rates of MAGE-1 and MAGE-3 genes. Therefore, MAGE-1 and MAGE-3 genes were closely related to the degree of malignancy and prognosis of the ovarian cancer; thus, the two could be used as independent prognostic indicators for ovarian cancer. The positive rate of BAGE was higher in ovarian cancer patients with ascites (*P *< 0.05), which was consistent with the finding of Hofmann et al. [3] that BAGE mRNA was positively expressed in 56% of ovarian cancer patients with ascites. These data suggested that BAGE may be associated with the formation and development of ascites. In addition, MAGE, GAGE and BAGE genes were expressed in ovarian cancer cell lines SKOV3, A2780 and COC1, which further demonstrated the role of these genes in ovarian cancer diagnoses. It was reported that the vaccine manufactured by using MAGE gene products had an acceptable toxicity and displayed a good effect on tumor response in the absence of other therapy [7]. The MAGE-3 gene has been used as a reliable indicator to detect melanoma cells in peripheral blood in the Gene Chip studies, as well as to monitor the infiltrative growth of tumor cells, thus, allowing for the determination of appropriate treatment interventions [8].

The products of the MAGE, BAGE and GAGE family genes are tumor-specific antigens that are highly expressed in different histological types of tumors. Therefore, these genes have drawn much attention for tumor immunotherapy. Currently, the most common application of MAGE, BAGE and GAGE genes in the ovarian cancer vaccine is the peptide vaccine and DC vaccine [9]. Although recent studies of these genes, especially the MAGE genes, have been conducted by domestic and international scholars and have resulted in new insight into these genes, our understanding of these genes is still insufficient. Due to the heterogeneity of tumor cells, the expression of MAGE, BAGE and GAGE family members may be varied in different types of ovarian tumors or in different parts of the same tumor, and it is very difficult to choose a target antigen for immunotherapy [10]. Through the present work, we believe that the question of whether MAGE, BAGE and GAGE genes could be useful as molecular markers and tumor-specific immunotherapy target sites for ovarian cancer still requires more in-depth investigation.

## Competing interests

The authors declare that they have no competing interests.

## Authors' contributions

SZ was the guarantor of integrity of the entire study, designed the experiment, carried out the RT-PCR method studies and drafted the manuscript. XZ designed the experiment, carried out the RT-PCR method studies and participated in manuscript preparation. HY participated in the design of the study and performed the statistical analysis. YY participated in literature research, data analysis and manuscript editing. All authors read and approved the final manuscript.

## Pre-publication history

The pre-publication history for this paper can be accessed here:

http://www.biomedcentral.com/1471-2407/10/163/prepub
